# Nutrigenetics and personalised/stratified approaches to the provision of dietary advice

**DOI:** 10.1186/2049-3258-72-S1-K4

**Published:** 2014-06-06

**Authors:** Anne-Marie Minihane

**Affiliations:** 1 Nutrition Department, Norwich Medical School, University of East Anglia (UEA), Norwich NR4 7TJ, UK

## 

Nutrigenetics refers to the interaction of genotype and diet composition to influence metabolism, health status and the risk of diet-related diseases. Gene variants influence food choice and appetite and therefore the intake of dietary components, their absorption, metabolism and tissue uptake along with their impact on molecular targets and physiological processes.

The first draft of the majority (~90%) of the sequence of the human genome was published in a *Nature* article entitled ‘*Initial sequencing and analysis of the human genome’* in 2001 [1] with the complete sequence (~99.7%) available in 2004 [2]. At the time such information was considered by many to be the panaceas and one of the greatest ever medical achievements, with genetic analysis likely to refine disease risk and prediction and the personalisation/stratification of interventions in order to afford maximum benefits.

Thirteen years on many consider progress based on the human genome to be limited, with the exception of the identification of the genetic basis of Mendelian (monogenic) disorders. Much of the estimated heritable component of disease risk and response to environmental change is unaccounted for. Based on available information, it appears that rather than being overestimated, the heritability is dark matter (i.e. it is real but we cannot see it yet), attributable to as yet undetected rare variants, or the underestimation of the impact of known variants. Genotype-diet-phenotype associations are not homogenous and influenced by a whole range of variables such as sex, ethnicity, drug use and other lifestyle variables which could in part account for the apparent ‘hidden heritability’ [3]

Specific examples of nutrigenetic interactions will be presented, in particular in the area of *APOE* genotype, along with some consideration of the practical, ethical, social and cost implications of the wider use of genetic information in public health and clinical practice.
